# Combined protective effect of zinc oxide nanoparticles and melatonin on cyclophosphamide-induced toxicity in testicular histology and sperm parameters in adult Wistar rats 

**Published:** 2017-07

**Authors:** Fereshte Torabi, Majid Malekzadeh Shafaroudi, Nourollah Rezaei

**Affiliations:** 1 *Immunogenetics Research Center (IRC), Faculty of Medicine, Mazandaran University of Medical Sciences, Sari, Iran, *; 2 *Department of Anatomy and Cell Biology, Faculty of Medicine, Mazandaran University of Medical Sciences, Sari, Iran.*

**Keywords:** Cyclophosphamide, Zinc oxid, Melatonin, Toxicity, Sperm

## Abstract

**Background:**

Cyclophosphamide (CP) has been known as an anticancer drug with several side effects on various organs such as a male reproductive system that can cause infertility.

**Objective:**

To evaluate the possible combined effects of zinc oxide nanoparticles (nZno) and melatonin (Mel) on sperm parameters and histopathological changes of the testis in CP-treated rats.

**Materials and Methods:**

42 adult male Wistar rats were divided into six groups. GI: control, GII: 60 mg/kg/wk CP, GIII and GIV, 10 mg/kg/wk Mel and 5mg/kg/wk nZno and GV: 5 mg/kg/wk nZno and 10 mg/kg/wk Mel were given 2 hr prior to CP injection, respectively,GVI: 5mg/kg/wk nZno and 10 mg/kg/wk Mel simultaneously. After 8 wk of treatment, rats were sacrificed and testis and epididymis were harvested for further evaluation.

**Results:**

The CP-treated group showed significant decreases in the body, testes and epididymis weights and sperm parameters (sperm count, viability, motility) with an increase abnormal sperms when compared with the control (p<0.001), as well as many histological alterations included decreased diameters of seminiferous tubules and Johnsen’s Testicular Score (with degeneration, desquamation, multi-nucleated giant cell formation), whereas combined treatment (GV), showed more protective effects on CP-induced reproductive system damage compared with groups III or IV (p<0.001).

**Conclusion:**

These results suggest simultaneous administration of Mel and nZno have more effectively protections against CP-induced reproductive damage than Mel or nZno alone.

## Introduction

Chemotherapy is one of the most common approaches to treat cancer, but it can cause various types of damage to normal living cells. The high frequency of cellular divisions that happens in the cells of the seminiferous epithelium makes the testis a highly sensitive organ to the chemotherapeutical drugs (1).

Cyclophosphamide (CP) is an alkylating agent used extensively as an antineoplastic and immunosuppressive drug. It is used to treat Hodgkin’s disease, lymphomas, leukemia, Wegener’s granulomatosis, severe rheumatoid arthritis, and lupus erythematosus. It is also used in combination with other drugs to treat breast cancer, leukemia, and ovarian cancer (2, 3). However, despite its wide range of application, CP also exhibit a wide range of adverse effects including reproductive toxicity in experimental animals and humans (4). 

Research shows that adult male patients treated with CP have indicated diminished sperm counts and a lack of spermatogenic cycles in their testicular tissue and long-term treatment with CP injuries progeny reduces the weight of the reproductive organs and impairs fertility. Thus, long-term side effects like the gonadal toxicity have become the main issue in the people treated with CP (5, 6). Although the exact mechanism of CP-induced testicular toxicity is still not clearly understood, oxidative stress and the generation of reactive oxygen species have been implicated in the pathophysiology of CP toxicity (7). In the recent years, concerns have been increased by the possible mutagenic effect of chemotherapy on spermatozoa. Previous studies have shown that treating rats with CP caused significant reduce in epididymal sperm count and motility and increases abnormal sperm rate (8, 9).

There are a number of agents of which the protective effects have been shown against CP-induced male reproductive toxicity; however, their safety and effectiveness for human use remain largely ambiguous. Among them, melatonin has the highest potential to not only safely counteract the adverse effects of CP, but also synergize its anti-cancer effects (10). Several investigators believe that melatonin’s antioxidant properties are due to its ability to scavenge Reactive Oxygen Species and increase cellular antioxidants (11, 12). 

On the other hand zinc (Zn) also known as an antioxidant with high conservation. Zn is an essential trace element required for maintenance of germ cells, the progression of spermatogenesis and regulation of sperm motility. In vitro, Zn exerts a direct protective effect in human spermatozoa by protecting against reduced motility and increased DNA fragmentation (13, 14). High concentration of zinc found in the testes and accessory sex glands show that it plays a crucial role in the reproductive system (15). Nanoparticles are materials with at least one dimension ≤100 nm, and this large surface-to-volume ratio results in unique characteristics compared to their corresponding bulk materials (16). Zinc oxide nanoparticles (nZno) are the new forms of materials with prominent biological properties and low toxicity that appears excessive potential to pass some of the barriers to efficient targeting of cells and molecules in many diseases (17). 

In a study, the effects of pinealectomy and melatonin administration and its relationship with zinc in rats was evaluated. Their findings showed that pinealectomy results in a decrease in zinc level in rats, and that melatonin administration to pinealectomized rats prevents the decrease in this parameter. In addition, long-term administration of melatonin to rats leads to an increase in zinc concentration and this is a noteworthy indicator of the correlation between melatonin and zinc (18). 

Since no scientific study on the effects of zinc along with melatonin has been reported, the present study was designed to investigate the possible combined effects of nano zinc oxide and melatonin on sperm parameters such as sperm count, motility, viability, abnormality and histopathological changes of the testis in CP-treated rats.

## Materials and methods

**Chemicals**

Cyclophosphamide (Endoxan Baxter®), Zinc oxide nanoparticles (Nanosany Company, particle size 10-30 nm [TEM], purity >99%), melatonin (M5250) and the other materials in this study were purchased from Sigma Chemicals Co.

**Animals and experimental design**

In this experimental study, 42 adult male Wistar rats (8-10 wk old, 200-250 gr) were purchased from Laboratory Animal Research Institute of Mazandaran University of Medical Sciences. The rats were kept under standard laboratory conditions (12 hr light/dark at 25-28^o^C) with a standard pellet diet and fed and tap water ad libitum during the treatment period. The animals were randomly divided into six experimental groups (n=7). Treatment groups were as follows: 

Group I served as a control receiving saline vehicle throughout the experiment. 

Group II (CP group): Injected with 60 mg/kg/wk CP (dissolved in saline)

Group III (Mel+ CP group): Injected with 10 mg/kg/wk Mel (dissolved in saline) followed by 60 mg/kg/wk CP, 2 hr later

Group IV (nZno+ CP group): Injected with 5 mg/kg/wk nZno (dissolved in saline) followed by 60 mg/kg/wk CP, 2 hr later

Group V (nZno+ Mel+ CP group): Injected with 5 mg/kg/wk nZno and 10 mg/kg/wk Mel followed by 60 mg/kg/wk CP, 2 hr later

Group VI (nZno+ Mel group): Injected with 5 mg/kg/wk nZno and 10 mg/kg/wk Mel simultaneously.

All treatments were applied Intra peritoneal and maintained for 8 wk. At the end of the experiment, the rats were weighed then under anesthesia by ketamine (200 mg/kg b.w.), their testes and epididymis were rapidly removed, and absolute weights of the epididymis and testes were measured and their weights relative to body weight were estimated. The right testis was used for histopathological examinations and right epididymis for sperm parameters analysis. 

**Sperm analysis**

Epididymal sperm analysis including motility, viability, count and abnormal morphology rates were done as described previously (19-21).

**Sperm motility**

The caudal part of epididymis was minced finely with scissors in a petri dish containing 1 ml of Ham’s F10 medium and incubated at 37^o^C for 20 min to allow the spermatozoa to swim out from the epididymal tubules. 10 µl of sperm suspension was put on a slide, and a coverslip was placed over the droplet. At least 10 microscopic fields were selected randomly at ×400 magnifications, and the percentage of motile spermatozoa was determined.

**Sperm viability**

A known volume of 10µl of sperm suspension was mixed with an equal volume of eosin-nigrosine And the slide was prepared. After drying. slides were observed using a light microscope with ×1000 magnification and the percentage of live sperms (colorless or light pink) and dead sperms (red or dark pink color) was determined. In total, 200 sperms were counted in each slide and viability percentages of sperm were calculated.

**Sperm count**

The sperm count was determined by hemocytometer. In brief, a 50 µl aliquot of epididymal sperm was diluted with 200 µl of distilled water and approximately 10 µl of this diluted sample was transferred to each of the counting chambers of the Neubauer haemocytometer which a stone cover slip had been placed on and was allowed to stand for 5 minutes in a humid chamber to prevent drying. The cells were sedimented during this time and counted with a light microscope at ×400.

**Sperm morphology**

A fraction of each suspension was then mixed (1:1) with 1% eosin Y in distilled water and 30 min later smears were made, allowed to dry in air, and were mounted under a coverslip. 200 sperm cells were examined per slide to determine the morphological abnormalities at ×1000 magnification. Any disorders in the morphology and structure of either head or tail were regarded as abnormal.

**Histopathological assay**

The right testis was taken immediately and fixed in alcoholic Bouin’s fluid for 24-48 hr, then processed into an automatic tissue processor system (SCILAB, England). The tissue processing included dehydration in ascending of grades alcohol 70, 80, 96% and absolute alcohol, then cleared in xylene followed by saturation in paraffin wax and finally, tissue blocks were prepared on paraffin block making Tissue Embedding Center (SCILAB, England). The 4-μm thick sections were obtained using a rotary microtome (Leica model RM 2145, Germany) and stained with hematoxylin and eosin method for microscopic examination.

**Morphometric study**

Seminiferous tubules diameter and germinal epithelial height of the 50 round or nearly round cross-sections of seminiferous tubules using an ocular micrometer of light microscopy (Nikon, Japan) were randomly measured in each animal and their means were calculated. The epithelial thickness was evaluated from the spermatogenic cells on the basement membrane through the sidelines cells of the tubules lumen.

**Spermatogenesis assessment**

To evaluate spermatogenesis, at least 100 seminiferous tubules were examined in each animal and quality of spermatogenesis in each tubule was scored according to the maturity of germ cells in the seminiferous tubules. Modified Johnsen score system uses a grade from 1-10 to each seminiferous tubule according to the following criteria, 10: complete spermatogenesis with many spermatozoa present, 9: slightly impaired spermatogenesis, many late spermatids, disorganized epithelium, 8: less than five spermatozoa per tubule, few late spermatids,7: no spermatozoa, no late spermatids, many early spermatids, 6: no spermatozoa, no late spermatids, few early spermatids, 5: no spermatozoa or spermatids, many spermatocytes, 4: no spermatozoa or spermatids, few spermatocytes, 3: spermatogonia only, 2: no germinal cells, Sertoli cells only, 1: no seminiferous epithelium. To calculate the Johnsen’s score, the sum of all scores was divided by the total number of seminiferous tubular sections (22). 

**Ethical consideration**

This experimental study was conducted with the approval of the local ethics committee on use and care for animal experiments at Mazandaran University of Medical Sciences. (Code 95-135). 

**Statistical analysis**

The obtained data were analyzed using SPSS version 18 (Statistical Package for the Social Sciences, version 18.0, SPSS Inc, Chicago, Illinois, USA). Results are presented as mean±SD, and all statistical comparisons were performed by means of one-way analysis of variance (ANOVA) followed by Tukey’s multiple comparison post hoc tests. p-value ˂0.05 was considered significant.

## Results

**Clinical signs and body weight changes**

The general effects of CP were narcosis, hunched posture, flaccid, low activity and a decreased appetite only seen in CP group animals. Compared to the control group, CP significantly decreased the body weight in CP and nZno+ CP groups, which may be ascribed to the decreased feed intake during the treatment (242.29±59.04 and 240.00±37.19 vs. 364.29±18.80 gr on the day of sacrifice; p<0.001). 

**Effects of nZno and Mel on reproductive organs weights**

As shown in table I, exposure of adult male rats to CP resulted in a significant decrease in the relative weights of the testis and epididymis compared to the control group. However, co-treatments with nZno and Mel caused a significant increase in the relative weights of testes and epididymis in comparison with CP group (p<0.001).

**Sperm examination findings**

**Figure 1:**
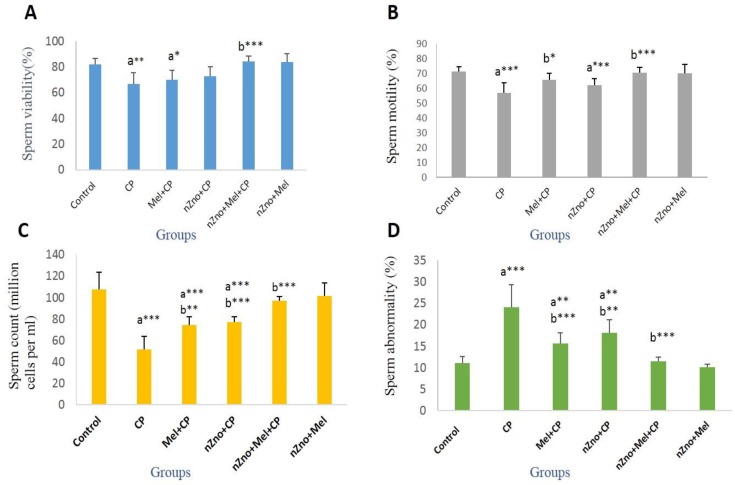
Sperm parameters in control and treated groups. (Mean±STD), Statistical significance is represented as follows: *p<0.05, **p<0.01, ***p<0.001, a: compared with control group; b: compared with CP group

The results of the sperm examinations are presented in figure 1, 2. Treatment of male rats with CP caused a significant decrease in the sperm viability, motility, count and increasing abnormal sperm rates, while co-administration of nZno and Mel compared to nZno or Mel alone more effectively protected against the CP-induced changes and minimized the toxic effects of CP (p<0.001).

**Histopathological findings**

Table II shows the Johnsen’s scores, seminiferous tubule diameter and germinal epithelium height for all the groups. At the end of the treatment, animals treated with CP showed a significant decrease in the mean of these parameters in comparison to the control group. On the other hand, these parameters in the nZno+ Mel+ CP group (with co-administration nZno and Mel) were significantly higher than Mel+CP and nZno+CP groups compared to CP-treated animals only (p<0.001 ).

Upon microscopic examination, animals in control and nZno +Mel groups (I and VI) revealed a normal testicular architecture and morphology with spermatogenic cells at different stages of development. Compared with control rats, CP treatment alone induced various histopathological alterations in the seminiferous tubules such as reductions in the numbers of germ cell in various stages of spermatogenesis,degeneration,desquamation, disorganization, multi-nucleated giant cell formation and significantly fewer spermatozoa in tubules. 

A detachment of spermatogenic cells, vacuolization and exfoliation of germ cells into the lumen of seminiferous epithelium were other features of the CP group. However, nZno or Mel alone ameliorated the CP- induced pathological lesions to a certain extent, but co-administration of nZno+Mel significantly attenuated the severity of CP-induced histopathological changes and impaired spermatogenesis (Figure 3 A-I).

**Table I:** Effects of zinc oxide nanoparticles, melatonin and cyclophosphamide on body and relative testis and epididymis weights of rats in control and treated groups

	**Control**		**CP**	**Mel+ CP**	**nZno+ CP**	**Mel+ nZno+ CP**	**Mel+ nZno**
Initial body weight (gr)	214.00±10.00		237.70±16.23	242.29±6.80	203.14±4.74	207.86±6.46	210.00±6.65
Final body weight (gr)	364.29±18.80		242.29±59.04^a^^***^	297.71±40.08^a^^*^	240.00±37.19 ^a^^***^	336.14±24.88^b^^**^	343.14±45.48
Right testis							
Absolute weight testis (gr)	1.54±1.75		1.15±1.37	1.32±0.09	1.20±0.11	1.42±0.11	1.50±0.13
Relative weight testis (gr)	0.71±0.08		0.50±0.06 ^a^^***^	0.54±0.04 ^a^^***^	0.58±0.04 ^a^^**^	0.68±0.06^b ^^***^	0.71±0.06
Left testis							
Absolute weight testis (gr)	1.57±1.70		1.21±0.14	1.36±0.11	1.22±0.11	1.46±0.08	1.52±0.16
Relative weight testis (gr)	0.73±0.08		0.52±0.08 ^a^^***^	0.55±0.05 ^a^^***^	0.59±0.04 ^a^^**^	0.70±0.04^b ^^***^	0.72±0.07
Right epididymis							
Absolute weight epididymis (gr)		0.60±0.07	0.43±0.12	0.51±0.05	0.48±0.11	0.57±0.05	0.62±0.10
Relative weight epididymis (gr)		0.28±0.04	0.19±0.04 ^a^^**^	0.20±0.02 ^a^^ *^	0.23±0.04	0.27±0.02^b^^**^	0.29±0.04
Left epididymis							
Absolute weight epididymis (gr)		0.63±0.07	0.44±0.13	0.51±0.06	0.44±0.09	0.61±0.07	0.65±0.08
Relative weight epididymis (gr)		0.29±0.04	0.19±0.04 ^a^^***^	0.21±0.02 ^a^^**^	0.21±0.04 ^a^^*^	0.29±0.03^b ^^***^	0.30±0.03

The data are expressed as mean ± SD (Standard deviation). Statistical significance is represented as follows:

nZno: nano zinc oxid

Mel: melatonin

P: cyclophosphamide

* p< 0.05,

** p< 0.01,

*** p< 0.001,

a : compared with control group;

b : compared with CP group

**Table II:** Johnsen’s score, seminiferous tubule diameter and germinal epithelium height in different groups

**Groups**	**Johnsen’s scores**	**STD (µm)**	**GEH (µm)**
Control	9.52 ± 0.10	295.14±5.69	98.64 ± 3.59
CP	7.45 ± 0.27^a^	207.00 ±11.07 ^a^	60.50 ± 2.43 ^a^
Mel+ CP	8.57± 0.39 ^a^^, ^^b^	249.03 ± 6.73 ^a^^, ^^b^	79.71 ± 2.56 ^a^^, ^^b^
nZno+ CP	8.45 ± 0.38 ^a^^, ^^b^	250.71 ± 17.32 ^a^^, ^^b^	82.25 ± 2.77 ^a^^, ^^b^
nZno+ Mel+ CP	9.18 ± 0.21 ^b^	287.28 ±14.32 ^b^	95.07 ± 1.92 ^a^^, ^^b^
nZno+ Mel	9.30 ± 0.25	297.00 ± 7.70	101.21 ± 3.36

The data are expressed as mean±SD. Statistical significance is represented as follows:

STD: seminiferous tubule diameter

GEH: germinal epithelium height

CP: cyclophosphamide

Mel: melatonin

nZno: nano zinc oxid

a p< 0.001 compared with control group

b p< 0.001 compared with CP group.

**Figure 2:**
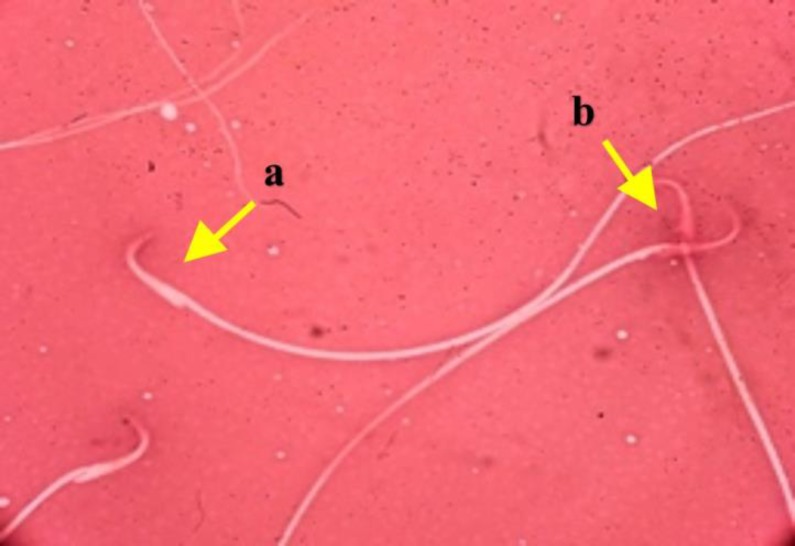
Evaluation of spermatozoa viability. a: Live sperm, with uncolored head. b: dead sperm, with red head (Eosin Y Nigrosine ×1000).

**Figure 3:**
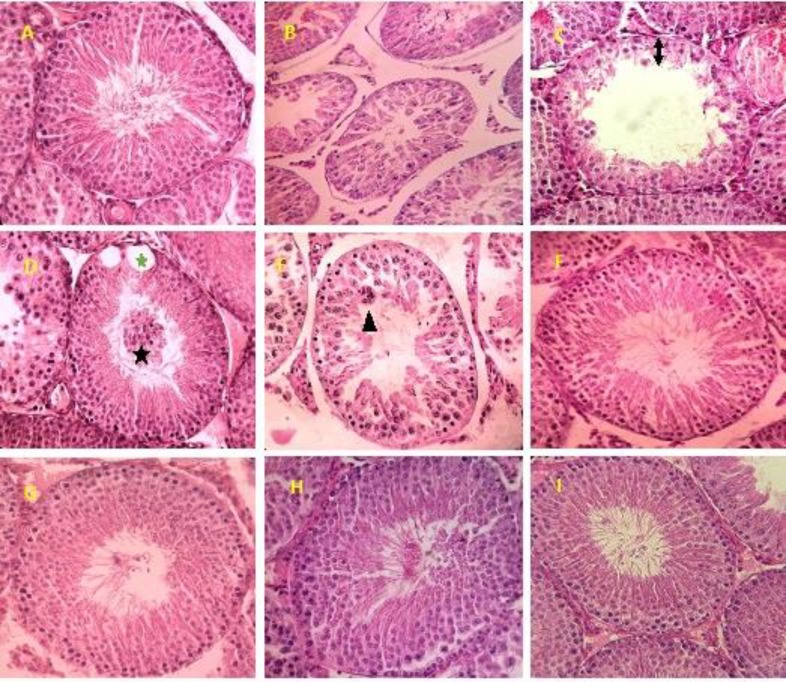
Normal testis of the control group, notice the normal spermatogenesis. **B-E: **Testes in cyclophosphamide (CP)-administered group showing degeneration, Germinal epithelium height reduction and incomplete spermatogenic series (Up down arrow), vacuolization (*) and exfoliation of germ cells into the lumen (*) and Multi-nucleated giant cell formation in some of the seminiferous tubule (arrow head). **F: **Mel+ CP group. **G:** nZno+ CP group. **H:** nZno+ Mel+ CP group, improvement of spermatogenesis of rats treated with CP plus simultaneous administration nZno and Mel. **I:** nZno+ Mel group. (H&E, ×400)

## Discussion

In this study, to determine the toxic effect of CP and possible protective roles of nZno and Mel in the reproductive system of male rats, we examined the sperm parameters and histopathological status of the testis. Our results showed a significant decrease in body weight, relative testis weight, and relative epididymis weight in animals treated with CP, consistent with results of previous studies (23, 25). However administration of nZno and Mel in group V significantly prevented the CP-induced reductions both in body weight and reproductive organ weight. According to Manson and kang changes in body weight are indicative of toxicity in mice, whereas the reduction in reproductive organ weight may be due to parenchymal atrophy, pathologic changes (degeneration and necrosis) and decrease in testosterone and sperm production in rats treated with CP and therefore effects of these agents on the testes may be due to their particular toxic effects on the target organ and not as a result of their general toxicity (8, 23, 26, 27). 

In our study, administration of CP significantly reduced epididymal sperm concentration, count, motility and increased abnormal morphology, confirming several studies (23, 28). The other studies have shown that the administration of CP once a week for 5 weeks caused oligospermia, azoospermia, testicular damage and germ cell toxicity in mouse (29, 30). According to the previous studies, increased morphological imperfection and production of abnormal sperms may also be a result of the direct toxicity of CP, because cellular DNA is a primary purpose of CP in its anti-cancer and toxic function thus, DNA damage may be responsible for the increased level of abnormal sperm shapes (5, 23). Another mechanism for CP toxicity on sperm morphology may also be peroxidation of polyunsaturated fatty acids in plasma membranes of spermatozoa by free radicals and low antioxidant capacity. It also has been reported that adenosine triphosphate (ATP) is an energy source for sperm motility and the reduced sperm motility following CP treatment may be the result of a disruption to ATP supply (13, 23).

The protective effect of melatonin against oxidative damage has been evaluated in both in vivo and in vitro studies (25). Melatonin prevented the CP- and cisplatin-induced increases in abnormal sperm. The rationale for the mechanism of the antimutagenic effects of melatonin is its ability to scavenge free radicals that cause oxidative DNA damage (23). In addition, melatonin indolent stimulatory effect on the production of ATP that according to our findings can improvethe motility of sperm. Zhang Showed that melatonin may potentially decrease testicular damage with improved histopathological changes in hyperlipidemic mice (31). 

Tuncer and co-worker*s *investigated histological effects of melatonin and zinc, alone or in combination, on rat testes and in contrast to other studies reported that 4-wk treatment with melatonin leads to histological and physiological impairments of the testis. Moreover, zinc supplementation might have a protective effect against the testicular damage caused by melatonin (15). In another study, effects of zinc and melatonin deficiency on rat testes were examined and their results indicated that testicular damage caused by zinc deficiency may be reduced by melatonin deficiency. Various studies have revealed the antioxidant and reproductive function of zinc. Zinc plays an important role in the physiology of spermatozoa and spermatogenesis because the zinc levels are very high in the male reproductive system and seminal fluid. It has been documented that zinc depletion causes atrophy of the seminiferous tubules and failure of spermatogenesis in rats (5, 32). Zn supplementation in rats attenuated various types of CP-induced sperm DNA damage. It also ameliorated CP-induced decrease of sperm count and plasma testosterone (10).

Several reports have shown that nanoparticles have a relatively higher toxicity compared to large size materials and it was reported that nZno has cytotoxic effects on some organs in time- and dose- dependent manners (5, 33). Talebi *et al* examined the effect of nZno on spermatogenesis in mice and showed that nZno has cytotoxic actions on testicular germ cells in a dose dependent manner (16). Also, Abbasalipourkabir and co-worker*s* 2015 investigated the effect of zinc oxide nanoparticles on adult male Wistar rats and indicated that the significant toxicity effects of nZno appear at concentrations above 50 mg/kg body weight of animals (34).

In the present study, nZno in the doses utilized (5 mg/kg) showed no toxic effect on spermatogenesis. In agreement with our results, Badkoobeh reported that administration of nZno prevented sperm damage in doxorubicin-treated adult male rats via an antioxidant mechanism (5). In another study, the chemoprotective efficacy of Nano-Selenium against CP-induced toxicity in tumor-bearing mice was investigated (35). In this study, epididymal sperm parameters including sperm count, motility, viability and percentage of abnormality showed a significant improvement with co-administration of nZno and Mel (group V) compared to CP-treated animals.

One study showed that the toxic effect of CP on spermatogenesis in rats was detected from day 7 after single administration (36). The particular sensitivity of the reproductive tissues to cyclophosphamide is due to the high proliferating activity. Morphometrical parameters such as seminiferous tubule diameter and germinal epithelium height can also give information about the testicular damage degree as a consequence of germ cell death (37). In the current study, reduced diameter of seminiferous tubules, germinal epithelium height, and Johnsen’s Testicular Score was observed in CP-treated rats compared to the control group. Also, histological observations in CP treated group demonstrated morphological alterations in seminiferous tubules such as degeneration, desquamation, disorganization, reduction in the number of spermatogenic cells, vacuolization, multinucleated giant cell formation and interstitial edema which is consistent with previous studies (19, 38). 

In this study, co-administration of nZno and Mel significantly improved CP-induced histopathological changes. Although nZno or Mel along with CP also ameliorated these changes in the testis to a certain extent but was less effective than the co-administration of nZno and Mel, which can be explained the synergistic effect of melatonin and zinc. Al-malki was evaluated the protective effect of lycopene alone or combined with Mel in inhibiting the oxidative stress and toxic effect of CP in rats and their results showed that supplementation of diet with lycopene and Mel provided the antioxidant defense with strong chemopreventive activity against CP-induced cytotoxicity (39). Also, Lu and co-workers observed that Zn (II)-curcumin compared to curcumin alone, proved more potent protection against the toxicity of CP in the testis as a result of the synergistic action of curcumin and Zn (13). The present study showed that co-administration of nZno and Mel to CP-treated rats significantly improved all studied parameters. In this mode usually, a combination with a low dose can intensify the effects of other compounds and reduce toxicity.

## Conclusion

In summary, the finding of our study indicates that exposure of adult male Wistar rats to CP had deleterious effects on both testicular histology and all male fertility parameters, while simultaneous administration of Mel and nZno cause protection against CP-induced reproductive damage which is more than the protection when Mel or nZno are used alone. That's probably due to the synergistic effect of zinc and melatonin. The relationship between zinc and melatonin is complex and not yet fully understood. Based on previous studies and our results, an apparent two-way relationship exists between zinc and melatonin. This observation should be further investigated.

## Note

***This article extracted from M.Sc. thesis. (Fereshte Torabi)***
